# Supplementary material for: Vibrio alginolyticus growth kinetics and the metabolic effects of iron
Source: Microbiol Spectr. 2023 Nov 15;11(6):e02680-23. doi: 10.1128/spectrum.02680-23 (PMC10714744; doi:10.1128/spectrum.02680-23)
Supplement: Supplemental material — Fig. S1 to S11; Tables S3 and S4. [file spectrum.02680-23-s0002.docx]

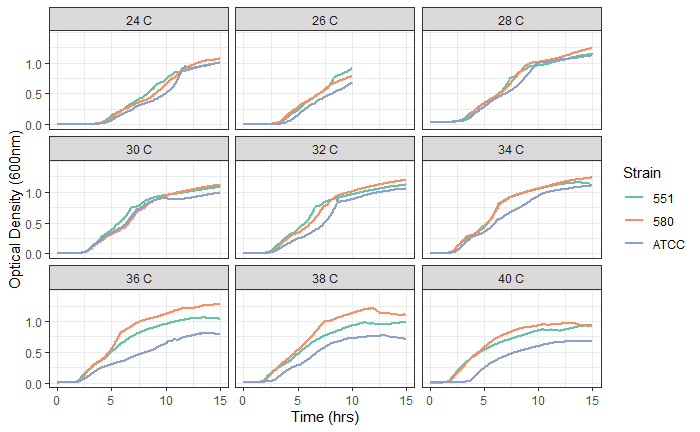


Figure S1: *V. alginolyticus* bacterial growth curves for temperature experimentation. All cultures grown in LBS 3% (w/v) NaCl broth at the indicated incubation temperature.


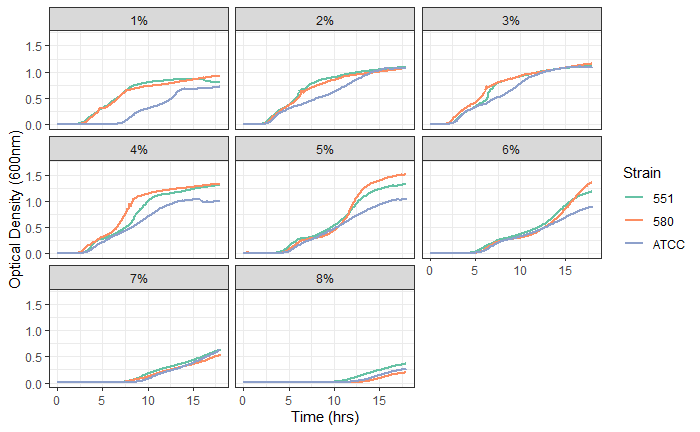


Figure S2: *V. alginolyticus* bacterial growth curves for salinity experimentation. All cultures grown in LBS broth amended to the indicated NaCl percent (w/v) at 30 ^o^C.


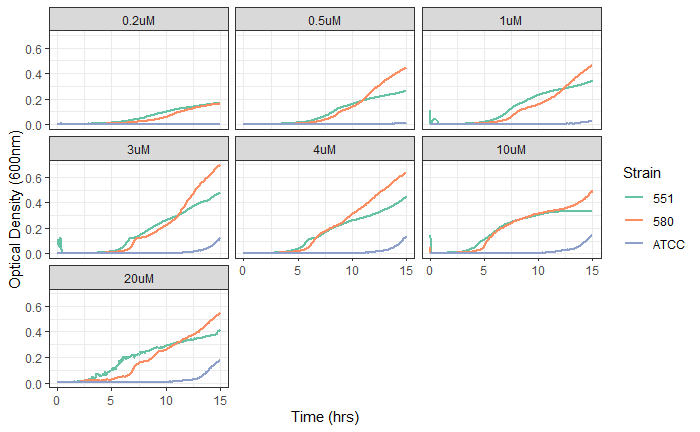


Figure S3: *V. alginolyticus* bacterial growth curves for iron experimentation. All cultures grown in VibFeL broth with an NaCl concentration of 3% (w/v) incubated at 30 ^o^C.


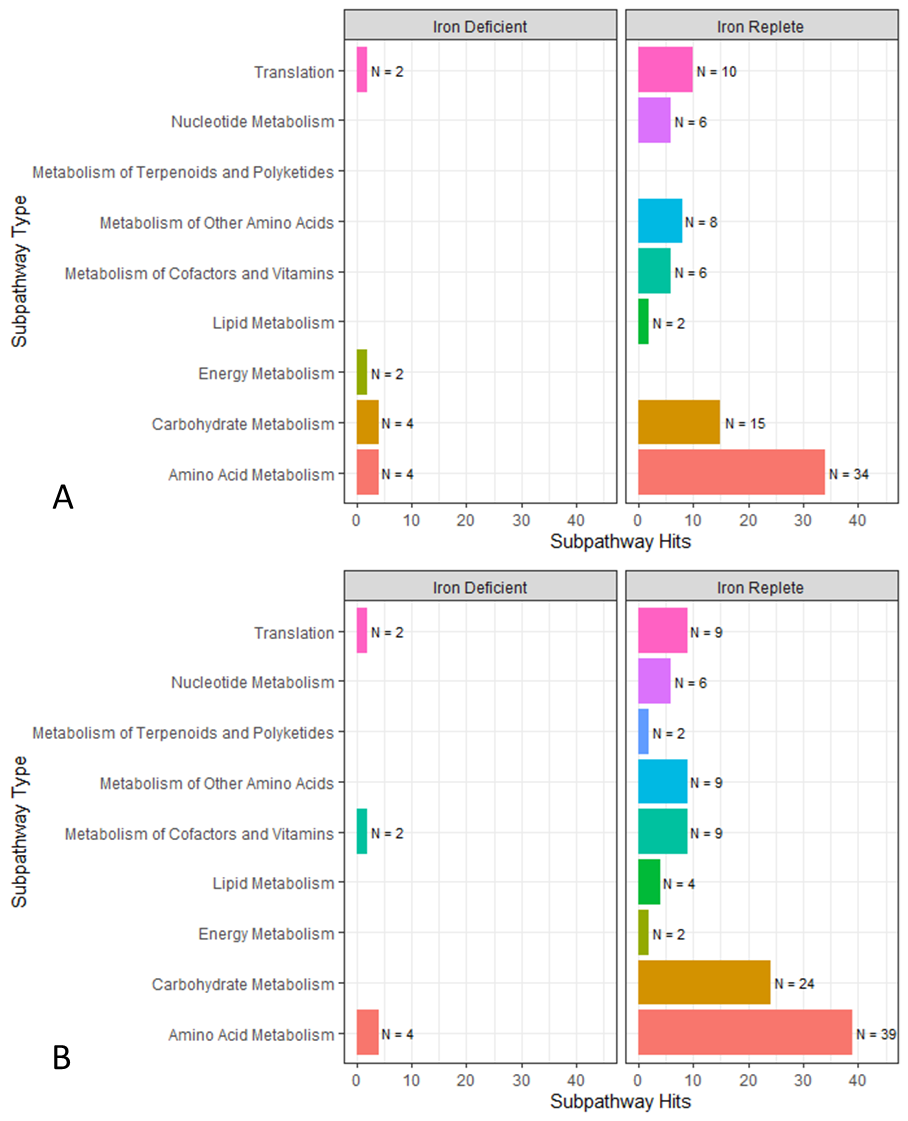


Figure S4: Upregulated metabolic pathways associated with iron replete and iron deficient *V. alginolyticus* endometabolite samples. The y-axis lists the detected KEGG subpathways and the x-axis shows the number of metabolite hits associated with each subpathway group. (A) Pathways comparisons for non-starved samples. (B) Pathway comparisons for starved samples.


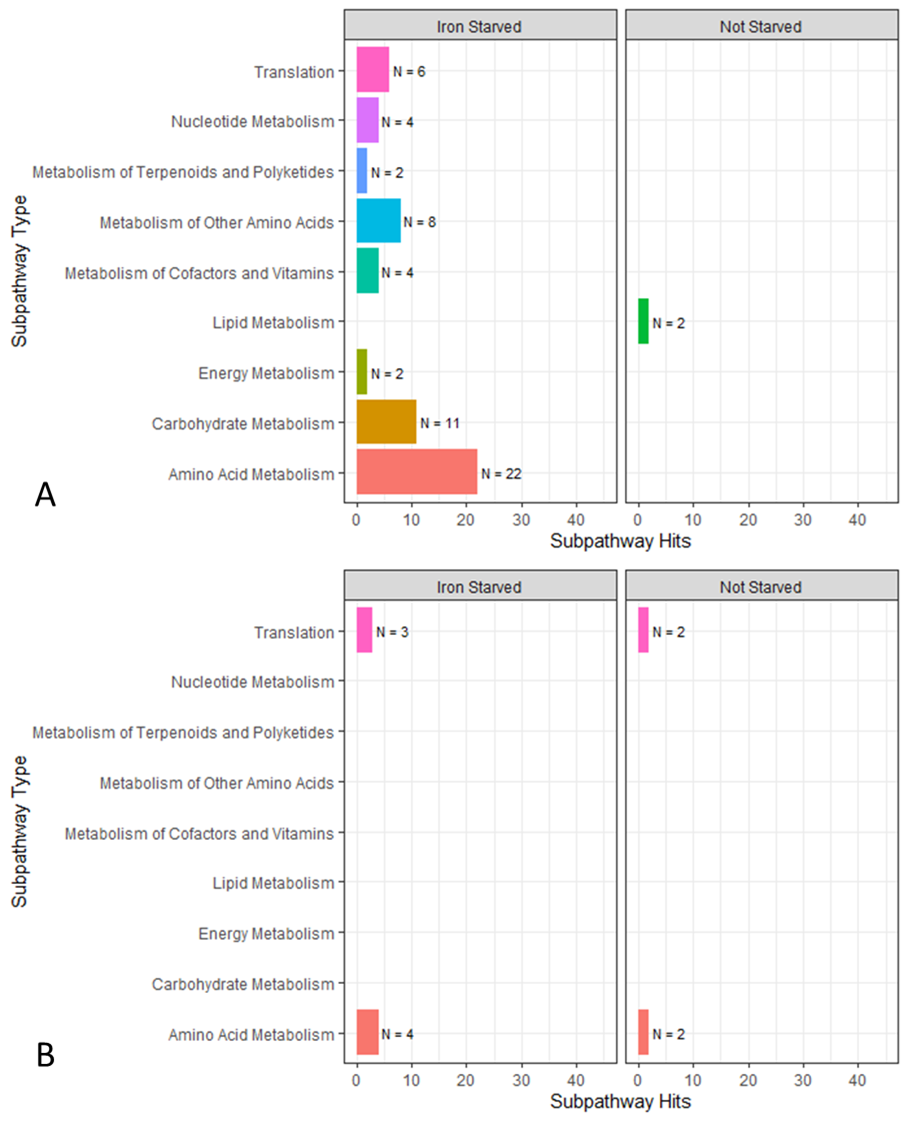


Figure S5: Upregulated metabolic pathways associated with iron starved and not starved *V. alginolyticus* endometabolite samples. The y-axis lists the detected KEGG subpathways and the x-axis shows the number of metabolite hits associated with each subpathway group. (A) Pathways comparisons for iron replete samples. (B) Pathway comparisons for iron deficient samples.


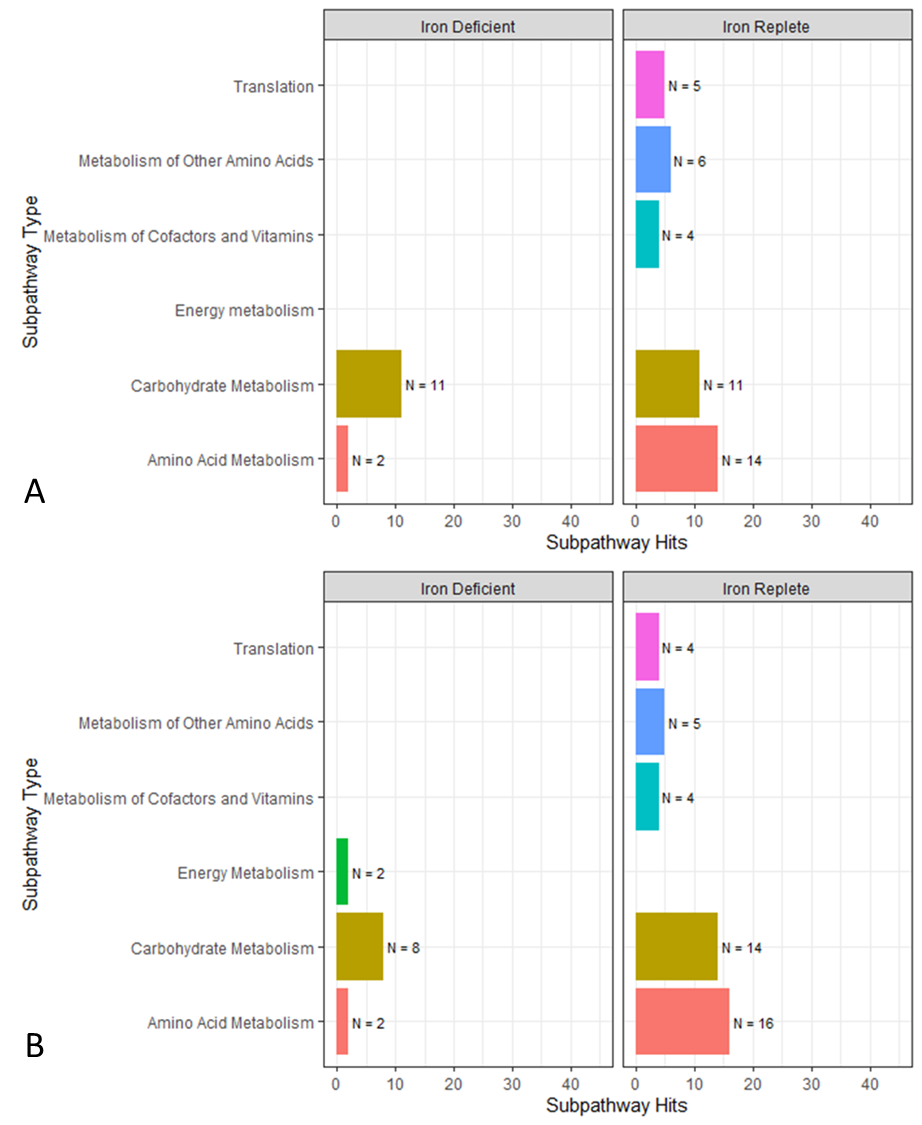


Figure S6: Upregulated metabolic pathways associated with iron replete and iron deficient *V. alginolyticus* exometabolite samples. The y-axis lists the detected KEGG subpathways and the x-axis shows the number of metabolite hits associated with each subpathway group. (A) Pathways comparisons for non-starved samples. (B) Pathway comparisons for starved samples.


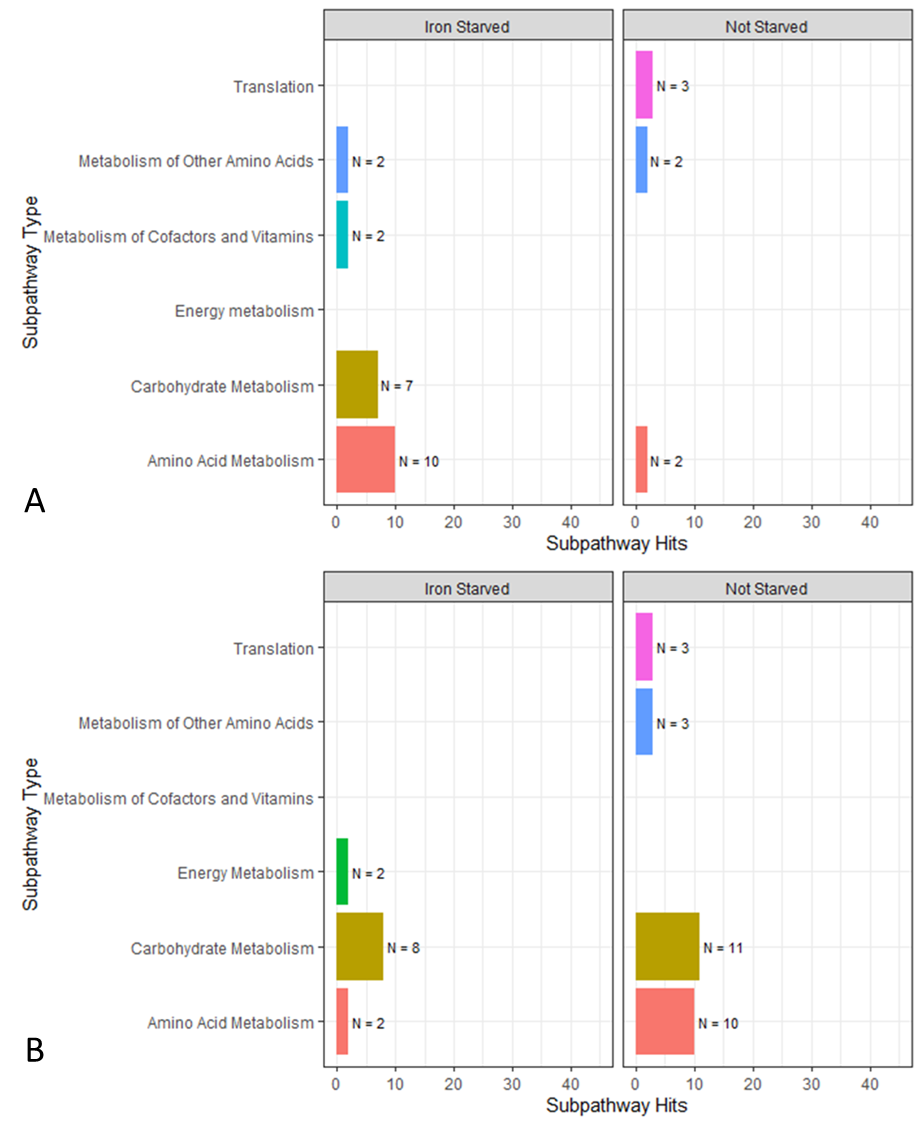


Figure S7: Upregulated metabolic pathways associated with iron starved and not starved *V. alginolyticus* exometabolite samples. The y-axis lists the detected KEGG subpathways and the x-axis shows the number of metabolite hits associated with each subpathway group. (A) Pathways comparisons for iron replete samples. (B) Pathway comparisons for iron deficient samples.


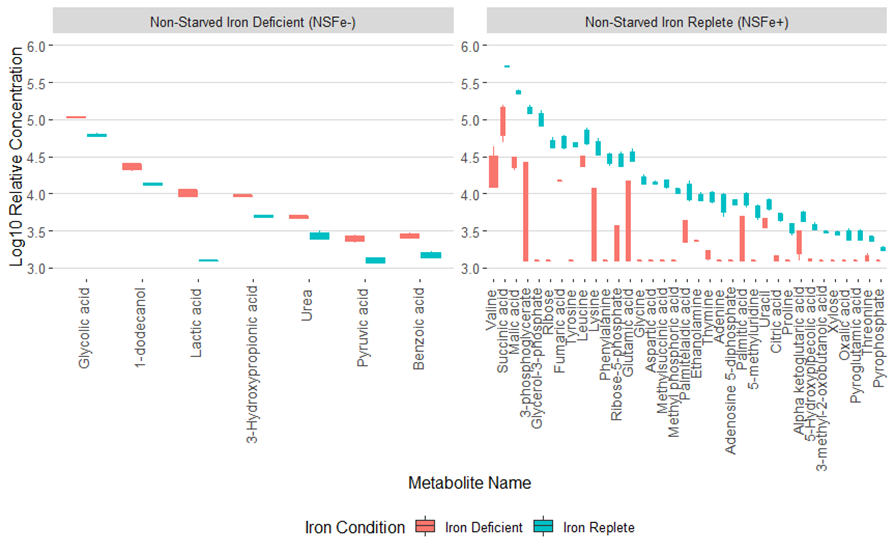


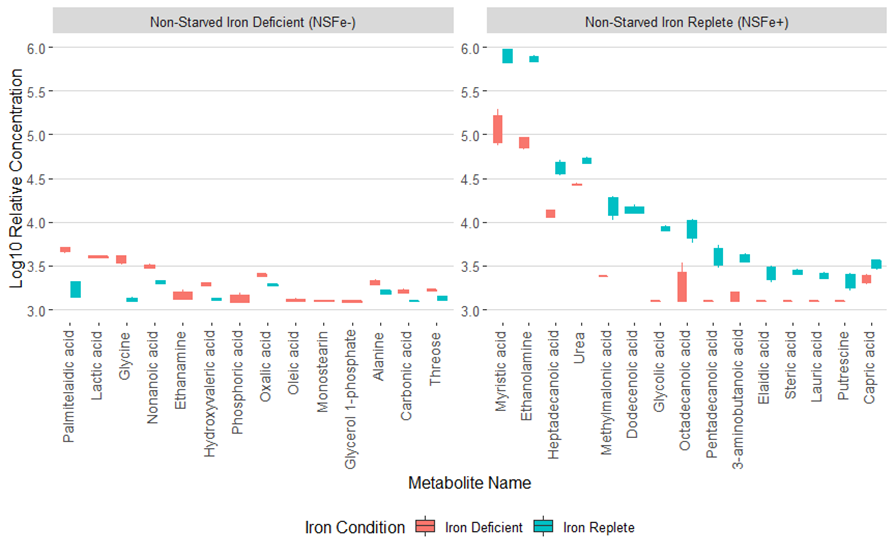


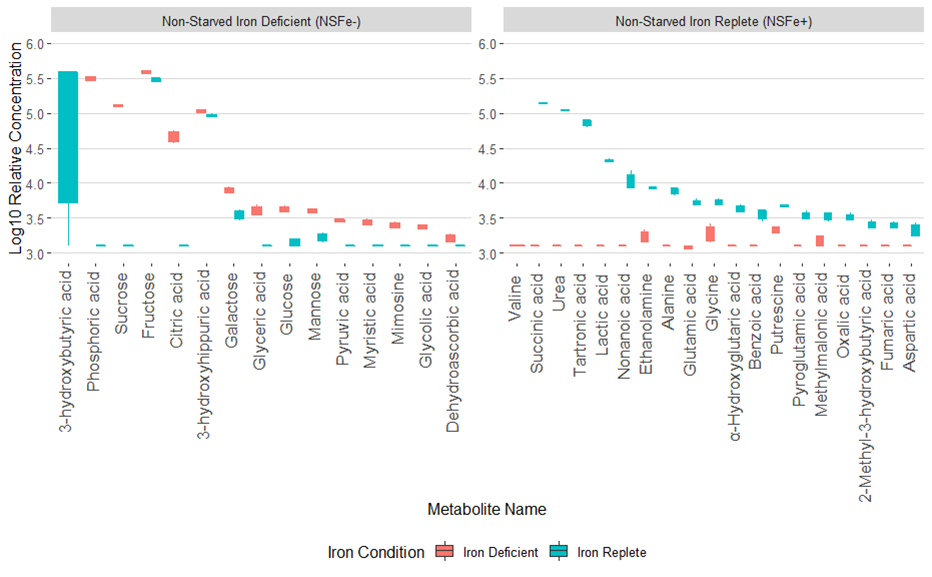


Figure S8: Up regulated metabolites identified from iron comparisons of non-starved cultures. (A) shows polar endometabolites, (B) shows non-polar endometabolites, and (C) shows exometabolites.


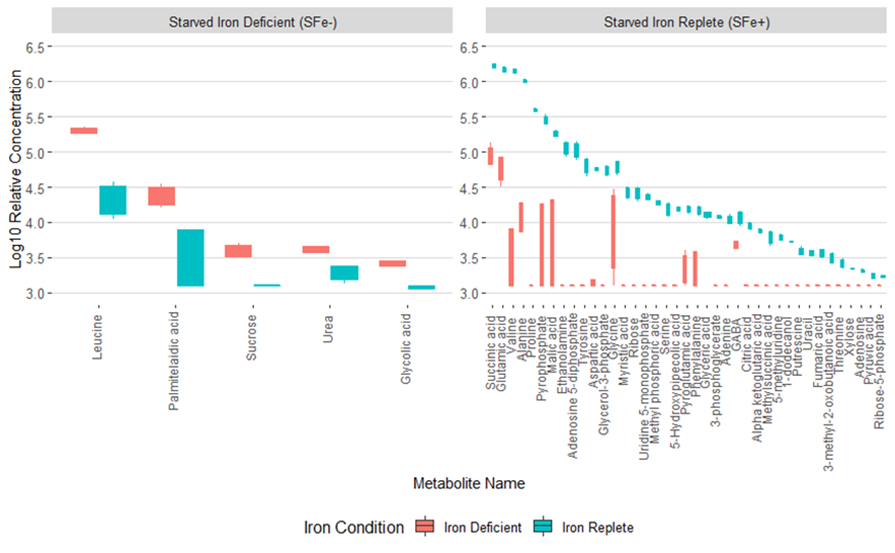

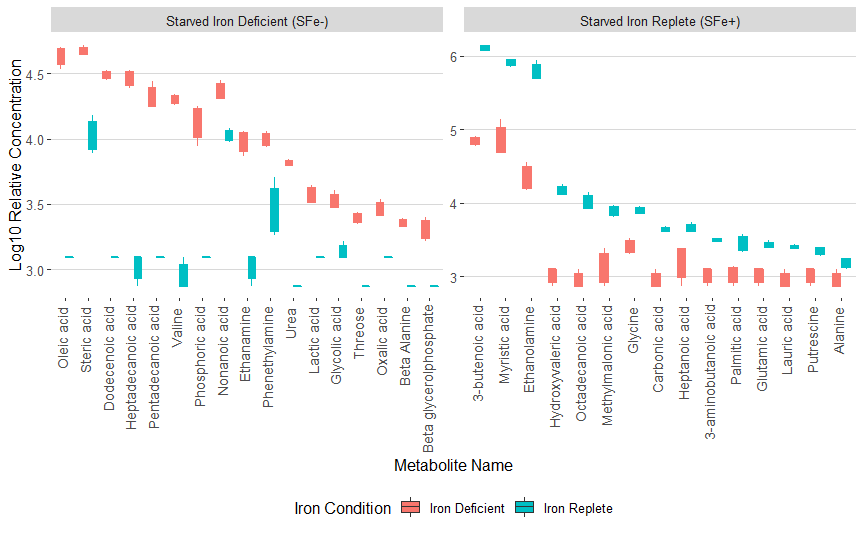


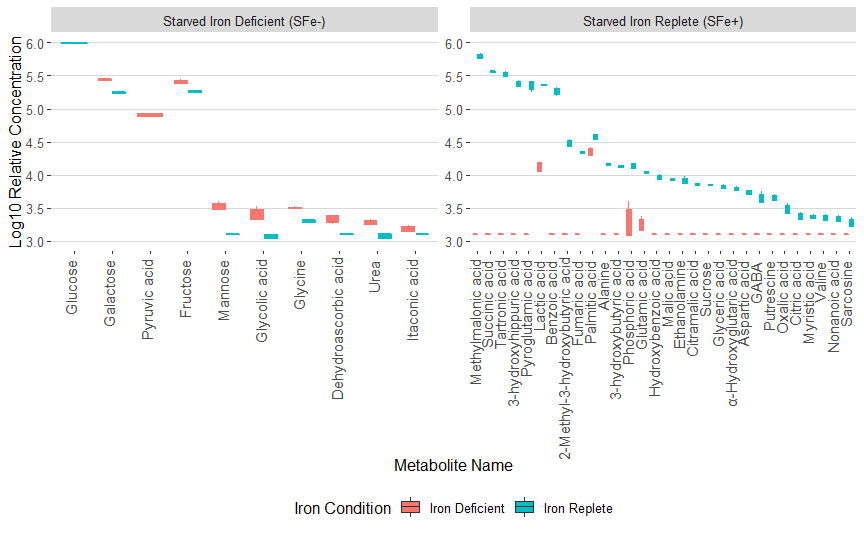


Figure S9: Up regulated metabolites identified from iron comparisons of starved cultures. (A) shows polar endometabolites, (B) shows non-polar endometabolites, and (C) shows exometabolites.


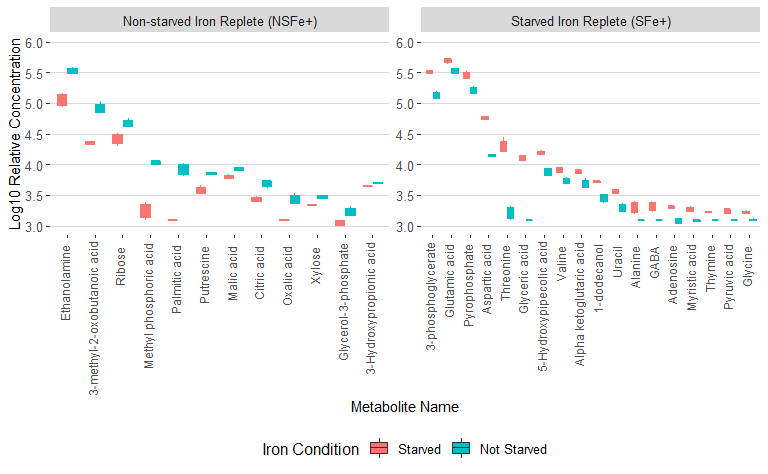


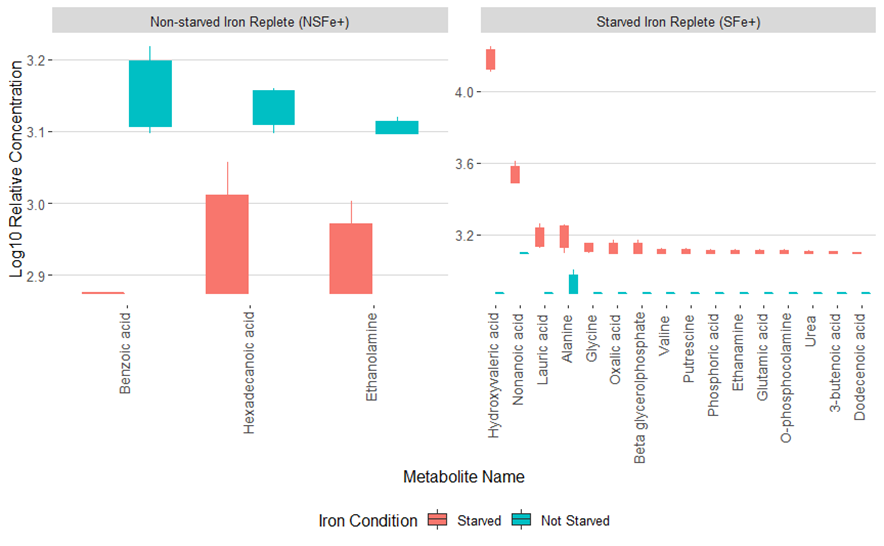


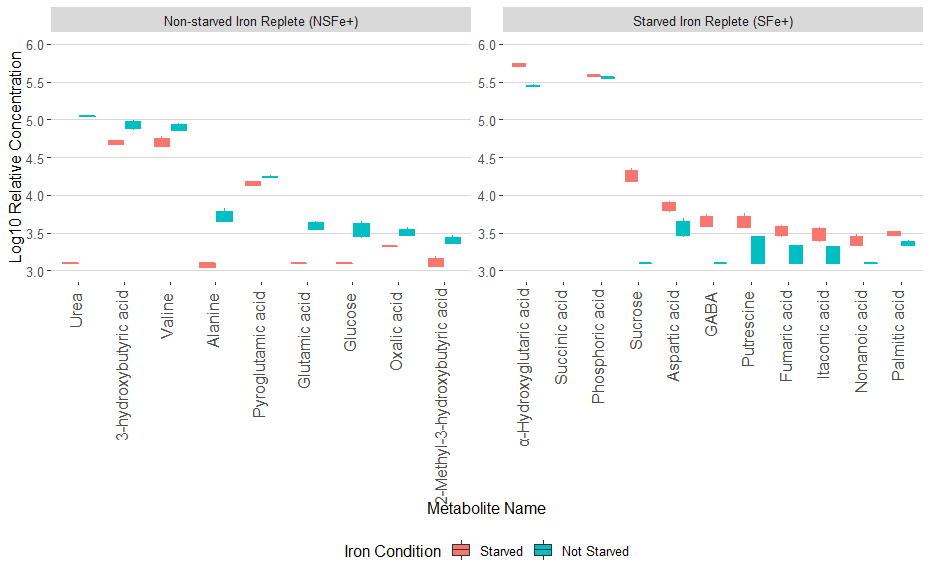


Figure S10: Up regulated metabolites identified from starvation comparisons of iron replete cultures. (A) shows polar endometabolites, (B) shows non-polar endometabolites, and (C) shows exometabolites.


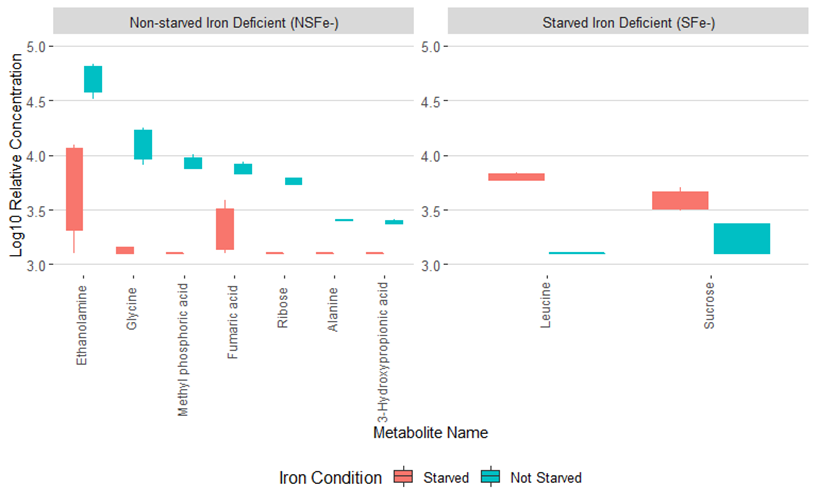


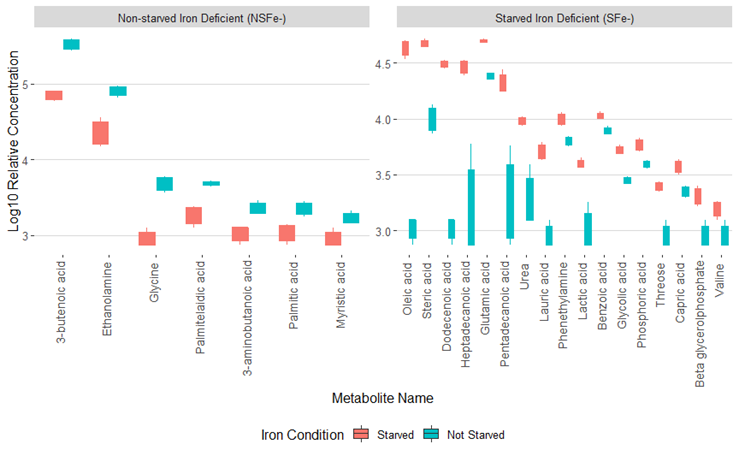


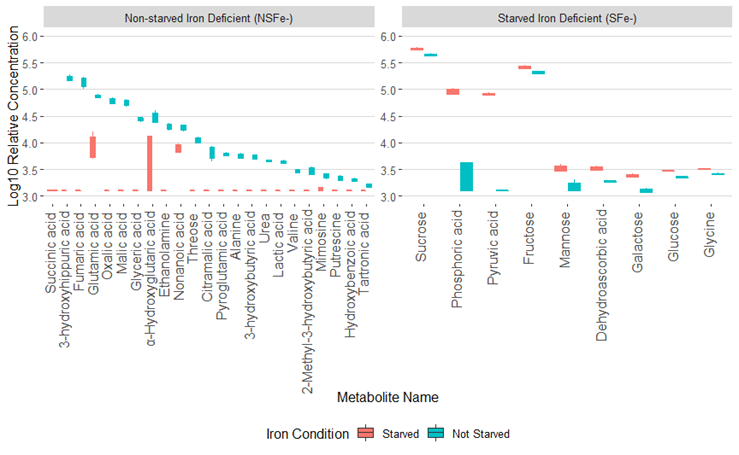


Figure S11: Up regulated metabolites identified from starvation comparisons of iron deficient cultures. (A) shows polar endometabolites, (B) shows non-polar endometabolites, and (C) shows exometabolites.

Table S3: Abiotic condition Kruskal Wallis significance test results^a^.

| Strain | Abiotic Metric | Growth Metric | Kruskal Wallis Test P-Value |
| --- | --- | --- | --- |
| JW16-551 | Temperature | Lag Phase | <0.001 |
| JW16-551 | Salinity | Lag Phase | <0.001 |
| JW16-551 | Iron Concentration | Lag Phase | <0.001 |
| JW16-551 | Temperature | Doubling Time | <0.001 |
| JW16-551 | Salinity | Doubling Time | <0.001 |
| JW16-551 | Iron Concentration | Doubling Time | <0.001 |
| JW16-580 | Temperature | Lag Phase | <0.001 |
| JW16-580 | Salinity | Lag Phase | <0.001 |
| JW16-580 | Iron Concentration | Lag Phase | <0.001 |
| JW16-580 | Temperature | Doubling Time | <0.001 |
| JW16-580 | Salinity | Doubling Time | <0.001 |
| JW16-580 | Iron Concentration | Doubling Time | <0.001 |
| ATCC 17749 | Temperature | Lag Phase | <0.001 |
| ATCC 17749 | Salinity | Lag Phase | <0.001 |
| ATCC 17749 | Iron Concentration | Lag Phase | <0.001 |
| ATCC 17749 | Temperature | Doubling Time | NA |
| ATCC 17749 | Salinity | Doubling Time | <0.001 |
| ATCC 17749 | Iron Concentration | Doubling Time | NA |

^a^Abiotic metrics tested for normality using a Shapiro Wilk test. All metrics demonstrated non-normal distributions (p-value > 0.05) thus Kruskal Wallis tests were applied to measure significance.

Table S4: Pairwise significance of abiotic conditions as reported using a Dunn post hoc test. P-values reported using a Holm-adjustment.

| Growth Metric | Abiotic Metric | Value Comparison^a^ | Dunn P-Value Strain JW16-551 | Dunn P-Value Strain JW16-580 | Dunn P-Value Strain ATCC 17749 |
| --- | --- | --- | --- | --- | --- |
| Lag Phase | Temperature | 24-26 | 1 | 1 | 1 |
| Lag Phase | Temperature | 24-28 | <0.001 | <0.001 | <0.001 |
| Lag Phase | Temperature | 24-30 | <0.001 | <0.001 | <0.001 |
| Lag Phase | Temperature | 24-32 | <0.001 | <0.001 | <0.001 |
| Lag Phase | Temperature | 24-34 | <0.001 | <0.001 | <0.001 |
| Lag Phase | Temperature | 24-36 | <0.001 | <0.001 | <0.001 |
| Lag Phase | Temperature | 24-38 | <0.001 | <0.001 | <0.001 |
| Lag Phase | Temperature | 24-40 | <0.001 | <0.001 | 0.335 |
| Lag Phase | Temperature | 26-28 | 0.055 | 0.266 | <0.001 |
| Lag Phase | Temperature | 26-30 | 0.460 | 0.657 | <0.001 |
| Lag Phase | Temperature | 26-32 | 0.061 | 0.312 | 0.019 |
| Lag Phase | Temperature | 26-34 | <0.001 | <0.001 | <0.001 |
| Lag Phase | Temperature | 26-36 | <0.001 | <0.001 | <0.001 |
| Lag Phase | Temperature | 26-38 | <0.001 | <0.001 | <0.001 |
| Lag Phase | Temperature | 26-40 | <0.001 | <0.001 | 1 |
| Lag Phase | Temperature | 28-30 | 1 | 1 | 1 |
| Lag Phase | Temperature | 28-32 | 0.704 | 1 | 1 |
| Lag Phase | Temperature | 28-34 | 0.110 | 0.009 | 0.02 |
| Lag Phase | Temperature | 28-36 | 0.009 | 0.04 | 0.016 |
| Lag Phase | Temperature | 28-38 | 0.001 | 0.001 | 1 |
| Lag Phase | Temperature | 28-40 | <0.001 | <0.001 | 0.02 |
| Lag Phase | Temperature | 30-32 | 1 | 1 | 0.839 |
| Lag Phase | Temperature | 30-34 | 0.067 | 0.006 | 0.341 |
| Lag Phase | Temperature | 30-36 | 0.006 | 0.030 | 0.345 |
| Lag Phase | Temperature | 30-38 | <0.001 | <0.001 | 1 |
| Lag Phase | Temperature | 30-40 | <0.001 | <0.001 | 0.009 |
| Lag Phase | Temperature | 32-34 | 0.530 | 0.061 | 0.002 |
| Lag Phase | Temperature | 32-36 | 0.081 | 0.180 | <0.001 |
| Lag Phase | Temperature | 32-38 | 0.020 | 0.015 | 1 |
| Lag Phase | Temperature | 32-40 | 0.005 | 0.004 | 0.359 |
| Lag Phase | Temperature | 34-36 | 1 | 0.887 | 0.903 |
| Lag Phase | Temperature | 34-38 | 1 | 1 | 0.151 |
| Lag Phase | Temperature | 34-40 | 0.696 | 1 | <0.001 |
| Lag Phase | Temperature | 36-38 | 1 | 1 | 0.135 |
| Lag Phase | Temperature | 36-40 | 1 | 1 | <0.001 |
| Lag Phase | Temperature | 38-40 | 1 | 1 | 0.021 |
| Lag Phase | Salinity | 1-2 | 1 | 1 | <0.001 |
| Lag Phase | Salinity | 1-3 | 1 | 0.503 | <0.001 |
| Lag Phase | Salinity | 1-4 | 1 | 1 | 0.006 |
| Lag Phase | Salinity | 1-5 | 0.536 | 0.725 | 0.141 |
| Lag Phase | Salinity | 1-6 | 0.013 | 0.515 | 0.840 |
| Lag Phase | Salinity | 1-7 | <0.001 | <0.001 | 0.684 |
| Lag Phase | Salinity | 1-8 | <0.001 | <0.001 | <0.001 |
| Lag Phase | Salinity | 2-3 | 0.599 | 1 | 0.777 |
| Lag Phase | Salinity | 2-4 | 0.451 | 0.993 | 0.615 |
| Lag Phase | Salinity | 2-5 | 0.021 | 0.558 | 0.301 |
| Lag Phase | Salinity | 2-6 | <0.001 | 0.037 | 0.012 |
| Lag Phase | Salinity | 2-7 | <0.001 | <0.001 | <0.001 |
| Lag Phase | Salinity | 2-8 | <0.001 | <0.001 | <0.001 |
| Lag Phase | Salinity | 3-4 | 1 | 1 | 0.967 |
| Lag Phase | Salinity | 3-5 | 0.160 | 0.067 | 0.173 |
| Lag Phase | Salinity | 3-6 | 0.002 | 0.002 | 0.006 |
| Lag Phase | Salinity | 3-7 | <0.001 | <0.001 | <0.001 |
| Lag Phase | Salinity | 3-8 | <0.001 | <0.001 | <0.001 |
| Lag Phase | Salinity | 4-5 | 1 | 0.467 | 0.774 |
| Lag Phase | Salinity | 4-6 | 0.189 | 0.049 | 0.132 |
| Lag Phase | Salinity | 4-7 | <0.001 | <0.001 | <0.001 |
| Lag Phase | Salinity | 4-8 | <0.001 | <0.001 | <0.001 |
| Lag Phase | Salinity | 5-6 | 0.866 | 1 | 1 |
| Lag Phase | Salinity | 5-7 | 0.013 | 0.005 | <0.001 |
| Lag Phase | Salinity | 5-8 | <0.001 | <0.001 | <0.001 |
| Lag Phase | Salinity | 6-7 | 0.327 | 0.151 | 0.015 |
| Lag Phase | Salinity | 6-8 | <0.001 | <0.001 | <0.001 |
| Lag Phase | Salinity | 7-8 | 0.017 | <0.001 | 0.011 |
| Lag Phase | Iron Content | 0.2-0.5 | <0.001 | <0.001 | NA |
| Lag Phase | Iron Content | 0.2-1 | <0.001 | <0.001 | NA |
| Lag Phase | Iron Content | 0.2-3 | <0.001 | <0.001 | NA |
| Lag Phase | Iron Content | 0.2-4 | <0.001 | <0.001 | NA |
| Lag Phase | Iron Content | 0.2-10 | <0.001 | <0.001 | NA |
| Lag Phase | Iron Content | 0.2-20 | <0.001 | <0.001 | NA |
| Lag Phase | Iron Content | 0.5-1 | <0.001 | 0.041 | NA |
| Lag Phase | Iron Content | 0.5-3 | <0.001 | <0.001 | NA |
| Lag Phase | Iron Content | 0.5-4 | <0.001 | <0.001 | NA |
| Lag Phase | Iron Content | 0.5-10 | <0.001 | <0.001 | NA |
| Lag Phase | Iron Content | 0.5-20 | <0.001 | <0.001 | NA |
| Lag Phase | Iron Content | 1-3 | 0.081 | 0.002 | NA |
| Lag Phase | Iron Content | 1-4 | 0.042 | <0.001 | NA |
| Lag Phase | Iron Content | 1-10 | <0.001 | <0.001 | NA |
| Lag Phase | Iron Content | 1-20 | <0.001 | <0.001 | NA |
| Lag Phase | Iron Content | 3-4 | 1 | 0.002 | 0.440 |
| Lag Phase | Iron Content | 3-10 | <0.001 | <0.001 | 0.116 |
| Lag Phase | Iron Content | 3-20 | <0.001 | 0.015 | <0.001 |
| Lag Phase | Iron Content | 4-10 | <0.001 | 0.281 | 0.379 |
| Lag Phase | Iron Content | 4-20 | <0.001 | 0.435 | <0.001 |
| Lag Phase | Iron Content | 10-20 | 0.701 | 0.062 | 0.013 |
| Doubling Time | Temperature | 24-26 | <0.001 | <0.001 | NA |
| Doubling Time | Temperature | 24-28 | <0.001 | <0.001 | NA |
| Doubling Time | Temperature | 24-30 | <0.001 | <0.001 | NA |
| Doubling Time | Temperature | 24-32 | <0.001 | <0.001 | NA |
| Doubling Time | Temperature | 24-34 | <0.001 | <0.001 | NA |
| Doubling Time | Temperature | 24-36 | <0.001 | <0.001 | NA |
| Doubling Time | Temperature | 24-38 | <0.001 | <0.001 | NA |
| Doubling Time | Temperature | 24-40 | <0.001 | <0.001 | NA |
| Doubling Time | Temperature | 26-28 | 0.243 | <0.001 | NA |
| Doubling Time | Temperature | 26-30 | <0.001 | <0.001 | NA |
| Doubling Time | Temperature | 26-32 | <0.001 | <0.001 | NA |
| Doubling Time | Temperature | 26-34 | <0.001 | <0.001 | NA |
| Doubling Time | Temperature | 26-36 | 0.834 | <0.001 | NA |
| Doubling Time | Temperature | 26-38 | <0.001 | <0.001 | NA |
| Doubling Time | Temperature | 26-40 | <0.001 | <0.001 | NA |
| Doubling Time | Temperature | 28-30 | <0.001 | 0.141 | NA |
| Doubling Time | Temperature | 28-32 | <0.001 | 0.084 | NA |
| Doubling Time | Temperature | 28-34 | <0.001 | <0.001 | NA |
| Doubling Time | Temperature | 28-36 | 0.270 | <0.001 | NA |
| Doubling Time | Temperature | 28-38 | <0.001 | <0.001 | NA |
| Doubling Time | Temperature | 28-40 | <0.001 | <0.001 | NA |
| Doubling Time | Temperature | 30-32 | 1 | <0.001 | NA |
| Doubling Time | Temperature | 30-34 | 1 | <0.001 | NA |
| Doubling Time | Temperature | 30-36 | <0.001 | <0.001 | NA |
| Doubling Time | Temperature | 30-38 | <0.001 | <0.001 | NA |
| Doubling Time | Temperature | 30-40 | <0.001 | <0.001 | NA |
| Doubling Time | Temperature | 32-34 | 1 | 0.001 | NA |
| Doubling Time | Temperature | 32-36 | <0.001 | <0.001 | NA |
| Doubling Time | Temperature | 32-38 | <0.001 | <0.001 | NA |
| Doubling Time | Temperature | 32-40 | <0.001 | 0.002 | NA |
| Doubling Time | Temperature | 34-36 | <0.001 | 0.002 | NA |
| Doubling Time | Temperature | 34-38 | <0.001 | 0.651 | NA |
| Doubling Time | Temperature | 34-40 | <0.001 | 0.689 | NA |
| Doubling Time | Temperature | 36-38 | <0.001 | 0.002 | NA |
| Doubling Time | Temperature | 36-40 | <0.001 | <0.001 | NA |
| Doubling Time | Temperature | 38-40 | <0.001 | 0.260 | NA |
| Doubling Time | Salinity | 1-2 | <0.001 | <0.001 | <0.001 |
| Doubling Time | Salinity | 1-3 | <0.001 | <0.001 | <0.001 |
| Doubling Time | Salinity | 1-4 | 0.113 | <0.001 | <0.001 |
| Doubling Time | Salinity | 1-5 | <0.001 | 0.001 | <0.001 |
| Doubling Time | Salinity | 1-6 | <0.001 | <0.001 | <0.001 |
| Doubling Time | Salinity | 1-7 | NA | NA | NA |
| Doubling Time | Salinity | 1-8 | NA | NA | NA |
| Doubling Time | Salinity | 2-3 | 0.112 | <0.001 | <0.001 |
| Doubling Time | Salinity | 2-4 | <0.001 | 0.002 | <0.001 |
| Doubling Time | Salinity | 2-5 | <0.001 | <0.001 | <0.001 |
| Doubling Time | Salinity | 2-6 | <0.001 | <0.001 | <0.001 |
| Doubling Time | Salinity | 2-7 | NA | NA | NA |
| Doubling Time | Salinity | 2-8 | NA | NA | NA |
| Doubling Time | Salinity | 3-4 | <0.001 | 0.010 | <0.001 |
| Doubling Time | Salinity | 3-5 | <0.001 | <0.001 | 0.004 |
| Doubling Time | Salinity | 3-6 | <0.001 | <0.001 | <0.001 |
| Doubling Time | Salinity | 3-7 | NA | NA | NA |
| Doubling Time | Salinity | 3-8 | NA | NA | NA |
| Doubling Time | Salinity | 4-5 | <0.001 | <0.001 | <0.001 |
| Doubling Time | Salinity | 4-6 | <0.001 | <0.001 | <0.001 |
| Doubling Time | Salinity | 4-7 | NA | NA | NA |
| Doubling Time | Salinity | 4-8 | NA | NA | NA |
| Doubling Time | Salinity | 5-6 | <0.001 | <0.001 | <0.001 |
| Doubling Time | Salinity | 5-7 | NA | NA | NA |
| Doubling Time | Salinity | 5-8 | NA | NA | NA |
| Doubling Time | Salinity | 6-7 | NA | NA | NA |
| Doubling Time | Salinity | 6-8 | NA | NA | NA |
| Doubling Time | Salinity | 7-8 | NA | NA | NA |
| Doubling Time | Iron Content | 0.2-0.5 | <0.001 | <0.001 | NA |
| Doubling Time | Iron Content | 0.2-1 | <0.001 | <0.001 | NA |
| Doubling Time | Iron Content | 0.2-3 | <0.001 | <0.001 | NA |
| Doubling Time | Iron Content | 0.2-4 | <0.001 | <0.001 | NA |
| Doubling Time | Iron Content | 0.2-10 | <0.001 | <0.001 | NA |
| Doubling Time | Iron Content | 0.2-20 | <0.001 | <0.001 | NA |
| Doubling Time | Iron Content | 0.5-1 | <0.001 | <0.001 | NA |
| Doubling Time | Iron Content | 0.5-3 | <0.001 | 0.919 | NA |
| Doubling Time | Iron Content | 0.5-4 | <0.001 | <0.001 | NA |
| Doubling Time | Iron Content | 0.5-10 | <0.001 | <0.001 | NA |
| Doubling Time | Iron Content | 0.5-20 | <0.001 | <0.001 | NA |
| Doubling Time | Iron Content | 1-3 | 0.307 | <0.001 | NA |
| Doubling Time | Iron Content | 1-4 | <0.001 | <0.001 | NA |
| Doubling Time | Iron Content | 1-10 | 0.013 | <0.001 | NA |
| Doubling Time | Iron Content | 1-20 | <0.001 | <0.001 | NA |
| Doubling Time | Iron Content | 3-4 | 0.012 | <0.001 | NA |
| Doubling Time | Iron Content | 3-10 | 0.004 | <0.001 | NA |
| Doubling Time | Iron Content | 3-20 | <0.001 | <0.001 | NA |
| Doubling Time | Iron Content | 4-10 | <0.001 | 0.595 | NA |
| Doubling Time | Iron Content | 4-20 | <0.001 | 0.303 | NA |
| Doubling Time | Iron Content | 10-20 | 0.511 | 0.046 | NA |

^a^Value comparison represents the two abiotic conditions compared. Temperatures reported in ^o^C, salinities reported in % (w/v) NaCl, and iron reported in µM.
